# Generation of Isogenic Induced Pluripotent Stem Cell Lines Derived From Individuals Mosaic for Loss of the Y Chromosome

**DOI:** 10.1002/alz70855_106554

**Published:** 2025-12-24

**Authors:** Holly N. Cukier, Yeunjoo E. Song, Lauren E. Coombs, Eugene Tang, Tianjie Gu, Brooke A. DeRosa, Derek M. Dykxhoorn, Jonathan L Haines, Margaret Pericak‐Vance, William S Bush, Anthony J Griswold

**Affiliations:** ^1^ Dr. John T. Macdonald Foundation Department of Human Genetics, University of Miami Miller School of Medicine, Miami, FL, USA; ^2^ John P. Hussman Institute for Human Genomics, University of Miami Miller School of Medicine, Miami, FL, USA; ^3^ Department of Population and Quantitative Health Sciences, Institute for Computational Biology, Case Western Reserve University, Cleveland, OH, USA

## Abstract

**Background:**

Mosaic loss of the Y chromosome (LOY) is a somatic, age‐related phenomenon associated with an increased risk of developing Alzheimer's disease (AD) by our group and others. While the underlying biological mechanism driving this risk remains unknown, genome wide association studies (GWAS) of LOY outcomes identified ∼150 loci involved in cell cycle regulation, mitotic pathways, and DNA damage response. These variants also predict X chromosome loss in women, suggesting that LOY is a marker of genomic instability in males that precedes the AD clinical phenotype.

**Method:**

To understand the mechanism by which LOY may contribute to AD risk, we sought to identify both affected and neurologically normal individuals with LOY from genotyping data using the MADloy program. We reprogrammed peripheral blood mononuclear cells (PBMCs) from three of these individuals and used the CytoTune iPS 2.0 Sendai Reprogramming Kit to create induced pluripotent stem cells (iPSCs) lines.

**Result:**

1,068 males with genotyping data were screened for loss of the Y chromosome; 41 (3.83%) had detectable LOY in the blood. As expected, LOY was positively correlated with the age of the individual at the time of sample collection. Three LOY samples (2 cognitively normal individuals and 1 individual with Alzheimer's disease) were chosen for iPSC reprogramming. Samples were independently shown to have LOY through either CNV TaqMan assays or whole genome sequencing data analyzed via the Control‐FREEC software. iPSC reprogramming was performed using Sendai virus. Clones were identified from each sample that either carried or were missing the Y chromosome, thereby generating isogenic pairs of cell lines.

**Conclusion:**

We have generated paired iPSC cell lines derived from AD and cognitively normal individuals that allow for explicit examination of the impact of LOY on AD‐relevant cell types.

